# Hydride Abstraction as the Rate-Limiting Step of the Irreversible Inhibition of Monoamine Oxidase B by Rasagiline and Selegiline: A Computational Empirical Valence Bond Study

**DOI:** 10.3390/ijms21176151

**Published:** 2020-08-26

**Authors:** Tana Tandarić, Alja Prah, Jernej Stare, Janez Mavri, Robert Vianello

**Affiliations:** 1 Division of Organic Chemistry and Biochemistry, Ruđer Bošković Institute, Bijenička Cesta 54, HR-10000 Zagreb, Croatia; tana.tandaric@irb.hr; 2 Laboratory for Computational Biochemistry and Drug Design, National Institute of Chemistry, Hajdrihova 19, SI-1001 Ljubljana, Slovenia; alja.prah@ki.si (A.P.); jernej.stare@ki.si (J.S.); janez.mavri@ki.si (J.M.); 3 Faculty of Pharmacy, University of Ljubljana, Aškerčeva Cesta 7, SI-1000 Ljubljana, Slovenia

**Keywords:** irreversible inhibition, monoamine oxidase, hydride transfer, antiparkinsonian drugs, neurodegeneration, flavoenzymes

## Abstract

Monoamine oxidases (MAOs) catalyze the degradation of a very broad range of biogenic and dietary amines including many neurotransmitters in the brain, whose imbalance is extensively linked with the biochemical pathology of various neurological disorders, and are, accordingly, used as primary pharmacological targets to treat these debilitating cognitive diseases. Still, despite this practical significance, the precise molecular mechanism underlying the irreversible MAO inhibition with clinically used propargylamine inhibitors rasagiline and selegiline is still not unambiguously determined, which hinders the rational design of improved inhibitors devoid of side effects current drugs are experiencing. To address this challenge, we present empirical valence bond QM/MM simulations of the rate-limiting step of the MAO inhibition involving the hydride anion transfer from the inhibitor α-carbon onto the N5 atom of the flavin adenin dinucleotide (FAD) cofactor. The proposed mechanism is strongly supported by the obtained free energy profiles, which confirm a higher reactivity of selegiline over rasagiline, while the calculated difference in the activation Gibbs energies of ΔΔ***G***^‡^ = 3.1 kcal mol^−1^ is found to be in very good agreement with that from the measured literature ***k***_inact_ values that predict a 1.7 kcal mol^−1^ higher selegiline reactivity. Given the similarity with the hydride transfer mechanism during the MAO catalytic activity, these results verify that both rasagiline and selegiline are mechanism-based irreversible inhibitors and offer guidelines in designing new and improved inhibitors, which are all clinically employed in treating a variety of neuropsychiatric and neurodegenerative conditions.

## 1. Introduction

Monoamine oxidases (MAOs) are flavoenzymes that metabolize a wide range of biogenic and dietary amines, including monoamine neurotransmitters, such as dopamine, serotonin, and adrenaline, in both the central nervous system and peripheral tissues [1]. Proper degradation of these molecules is responsible for the stable function of synaptic neurotransmission, resulting in a healthy brain condition. Since different concentrations of these small monoaminergic neurotransmitters influence our mood and emotions, as well as the control of motor, perceptual, and cognitive functions, any imbalance in their concentration leads to the development and progression of a number of, if not all, neuropsychiatric and neurological diseases.

There are two known isoforms of the MAO enzyme—MAO A and MAO B [2,3]—which share around 70% sequence identity and the same FAD cofactor covalently attached at a conserved cysteine residue [4]. Their active site cavities are placed deep inside the enzyme, and differ in volume and shape between isoforms [5], which results in different substrate and inhibitor affinities [6]. The chemical reaction catalyzed by both MAO isoforms involves the oxidation of the amine moiety via oxidative cleavage of the substrate α-CH bond which consequently generates an imine intermediate. This step is accomplished by the reduction of FAD to FADH_2_ that is followed by reoxidation back to FAD with molecular oxygen and a simultaneous H_2_O_2_ release. The imine intermediate is hydrolyzed nonenzymatically to the corresponding aldehyde and ammonia (with primary amines) or a substituted amine (from secondary amines) [7]. The overall catalytic MAO reaction is given as:

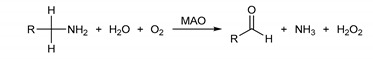


All products of the MAO-mediated reactions are highly reactive species, and include a number of potentially neurotoxic agents [8,9,10]. Because of that, constant excessive activity of MAO enzymes may lead to mitochondrial damages which lead to the cell damage and dysfunction, resulting in neurodegenerative disturbances. These premises are the main reason why MAO inhibition is recognized as an important tool in the therapy of various psychiatric and neurological diseases, ranging from mood disorders to Parkinson’s (PD) [11] and Alzheimer’s diseases (AD). Additional studies on MAO knock-out mice [12,13] have revealed that the inactivation of this enzyme produces a number of functional and behavioral changes, some of which are interesting for therapeutical use. The modulation of the brain and behavior induced by MAO inhibitors [14] made the design of new and more potent MAO inhibitors—one of the central topics of interest for both academia and industry over the last 60 years [15,16,17,18,19,20].

**Figure 1:**
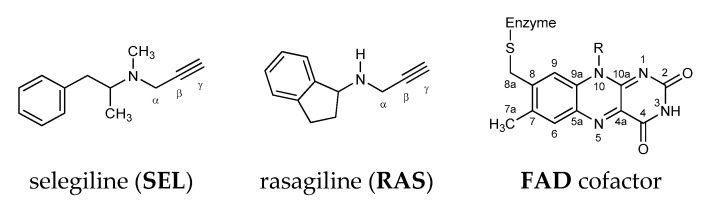
Chemical structure of the MAO B irreversible inhibitors and the FAD cofactor discussed in this work. The relevant atom labeling used throughout the text is also indicated.

First discovered MAO inhibitors were the nonselective reversible inhibitors ipronazid [21] and phenelzine, yet these compounds were shown to be associated with diverse adverse effects, including fatal liver toxicity [22] and the so-called “cheese reaction”, consisting of severe, potentially lethal hypertensive crises following the consumption of foods rich in amine tyramine [23], such as cheese and red wine. These side effects are mainly produced by intestinal MAO A inhibition, since the tyramine oxidation occurs exclusively by intestinal MAO A, which prompted research to develop and characterize selective inhibitors. Along these premises, selective MAO B inhibitors, such as selegiline (SEL) and rasagiline (RAS) (Figure 1), do not have such high incidence for fatal side effects, which warrants their use without the restriction of a low-tyramine diet [24].

Inhibiting MAO B prolongs the half-life of dopamine in neurosynapses and extends its neurotransmission effect for the improvement of motor symptoms. In addition, it also prevents the MAO B-mediated oxidative damages in neurons [25,26], further prompted by the fact that more than 80% of MAO in the human brain is of the B subtype [27]. Despite the facts listed above, MAO B inhibition drugs are still connected with significant adverse effects [27,28]. Furthermore, it is worth stressing that these two drugs are used only in symptomatic therapy, and they do not treat the cause of disease as dopaminergic agonists and L-DOPA. These facts underline the necessity for the design of new and more potent compounds devoid of any side reactions.

The majority of MAO inhibitors in the current use are mechanism-based irreversible inhibitors [11,29]. These drugs form a covalent bond with the enzyme, preventing further catalytic activity, which is the most successful way of inhibiting MAO B in vivo [30,31]. Selegiline (L-deprenyl, SEL) was the first such irreversible drug to be commercially used and has been on the market since the late 1980s [32]. Its adduct with the N5 atom on FAD has been identified both chemically [33] and in the crystal structure of the inhibited MAO B [34,35]. However, SEL is linked with a variety of side effects, mainly caused by its major L-amphetamine-like metabolite, which can cause appetite suppression, insomnia, and increased irritability [36,37]. In addition to that, amphetamines inhibit dopamine’s transport to vesicles [38,39], which potentiate autooxidation in the cytoplasm [40]. This process provides an additional source of the reactive oxygen species.

On the other hand, its propargyl analogue rasagiline (RAS) [41] was introduced to the market in the first decade of the last century. RAS features the same mechanism of action, but, unlike SEL, it has a much more favorable degradation inside of the human body. Not only are its metabolites linked with significantly less side effects, they also behave in a neuroprotective way [42,43]. The main metabolic product of RAS, 1-aminoindane, is proven to increase neuron life span, thus allowing a lower daily dose. All these reasons explain why RAS is widely used nowadays.

Clinical practice confirmed that propargylamine MAO B inhibitors are effective and safe medications that provide symptomatic benefits for PD patients from early to late stages of disease [44]. Because of this reason, a broad spectrum of therapeutic possibilities has been developed. Solutions such as the transdermal administration, as well as prodrugs which are converted to active inhibitors by brain enzymes that avoid problems with the lipophilicity, are promising directions for the future.

Over recent years, researchers developed many classes of potent and more selective compounds [45,46,47,48,49,50]. Additionally, many efforts were involved in understanding structural differences between MAO isoforms [51] that affect their selectivities. However, for a rational prediction of more effective systems, the details of a precise chemical mechanism of the inhibition reaction are crucial, and those are still missing. This would enable researchers to design mechanism-based drugs as transition-state analogues that are likely to improve the selectivity and efficacy in neurodegenerative diseases, which might allow for the use of lower therapeutic doses, thus strongly diminishing possible adverse effects.

**Figure 2:**
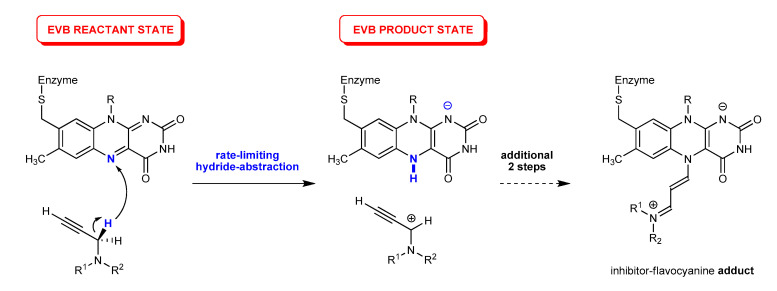
The initial rate-limiting step and the corresponding Empirical Valence Bond (EVB) states for the irreversible inhibition of MAO enzymes with propargylamine inhibitors based on a direct hydride abstraction studied here, which ultimately results in the inhibitor–FAD flavocyanine adduct as shown in ref. [52].

In our recent work [52], we performed molecular dynamics simulations [53] to investigate the binding of SEL and RAS within the MAO B active site and identified residues predominantly responsible for the successful binding. In addition, the calculated MM-GBSA binding free-energies effectively reproduced a trend in the measured ***K***_i_ and IC_50_ data, and thus confirmed that SEL binds better due to its bigger size and flexibility, allowing it to optimize hydrophobic C–H∙∙∙π and π∙∙∙π interactions with residues throughout both of the enzyme’s cavities, particularly with FAD, Gln206 and four active site tyrosines (Tyr435, Tyr398, Tyr60, Tyr326). In this way, SEL is able to overcome a larger ability of RAS to form hydrogen bonds that only position it in less reactive orientations for the subsequent inhibition reaction. More importantly, this was followed by the quantum mechanics (QM) cluster calculations [54] at the DFT level, which revealed that the MAO inactivation proceeded through a three-step reaction, where, in the rate-limiting first step, the MAO enzyme used the FAD’s N5 atom to abstract a hydride anion from the inhibitor’s α-CH_2_ group (Figure 2), being in a full analogy with the MAO catalytic mechanism [55,56,57,58], thus confirming that both SEL and RAS are mechanism-based inhibitors. The complete reaction profile led from the bound inhibitors to the corresponding N5(FAD)-adducts, the latter being in excellent agreement with the crystallographic data on the inhibited enzyme, and further confirmed a better reactivity of SEL through both its lower activation barrier and higher overall exergonicity [52]. Still, although the obtained relative difference in the activation free energies among inhibitors of ΔΔ***G***^‡^ = 1.2 kcal mol^−1^ was found to be in excellent agreement with the measured ***k***_inact_ values, which predicts a difference of ΔΔ***G***^‡^_EXP_ = 1.7 kcal mol^−1^, the absolute values of Δ***G***^‡^(SEL) = 27.9 kcal mol^−1^ and Δ***G***^‡^(RAS) = 29.1 kcal mol^−1^ clearly exceeded those predicted experimentally, although these were significantly lower than those calculated for several alternative mechanistic scenarios. Still, the overestimated barriers are most likely due to the known limitations of the employed cluster calculations that consider only a truncated part of the enzyme; while the rest of the enzyme environment was accounted through the polarized continuum approximation [59,60,61,62,63,64,65,66], requiring a fully featured treatment of the solvated enzymatic surroundings, which is known to significantly affect the active site, mainly via electrostatic interactions. To overcome these, here, we utilized the multiscale QM/MM approach, where the quantum subsystem was described by the Empirical Valence Bond (EVB) methodology [67] in conjunction with an all-atom classical representation of the hydrated enzyme. The purpose of this work was to attain a much better absolute agreement in the calculated kinetic parameters with experimentally measured data, thus further confirming the validity of the proposed hydride transfer mechanism in the case of the clinically employed inhibitors selegiline and rasagiline.

## 2. Results and Discussion

Selegiline is a weak base with the experimentally determined p***K***_a_ value of 7.44 for the protonation of its amino group in the aqueous solution [68]. This indicates that, under physiological conditions of pH = 7.4, selegiline assumes a practically equal population of its unionized and monocationic protonation states. While we were not able to locate the corresponding literature data for rasagiline, we can assume that its p***K***_a_ value should be roughly the same. Our earlier mechanistic proposal required unionized inhibitors to undergo the MAO inhibition [52], since it is significantly easier to detach the hydride anion from the neutral system and generate the carbocation, than it is from the already monocationic system that is transformed into dicationic compounds upon the hydride abstraction. This is justified considering the mentioned p***K***_a_ value for selegiline being close to the physiological pH value, but also knowing the hydrophobic nature of the MAO active site, which was clearly demonstrated experimentally [5,34] and confirmed computationally [69]. Once placed within such a hydrophobic environment, the amino groups within investigated inhibitors will likely experience a reduced basicity making their unionized analogues the predominant protonation forms prior to reacting with the FAD cofactor. Even if that would not be the case, such a deprotonation can easily be achieved by the active site water molecules with a cost of only a few kcal mol^−1^ [69,70]. In this context, we utilized neutral SEL and RAS in all subsequent calculations, and our DFT results with implicit SMD solvation for their aqueous phase reactivity are presented in Table 1.

In a reference reaction between each inhibitor and lumiflavin in water (Table 1), the investigated hydride abstraction reaction was a one-step process, which started from the unionized reactants and resulted in the carbocationic inhibitor at the C(α) position and the anionic cofactor featuring a newly formed N(5)–H bond (Figure 2). Interestingly, the reaction was kinetically slightly more favorable for RAS, as its activation free energy was, by ΔΔ***G***^‡^ = 0.51 kcal mol^−1^, lower than that for SEL. In the transition state structure, the transferring hydride anion was placed between the leaving α-carbon and the accepting N5 atom, with bond distances of 1.45 and 1.22 Å for RAS, respectively, being similar at 1.43 and 1.23 Å in SEL, in the same order. Specifically, this resulted in the activation free energy for this process of 30.6 kcal mol^−1^ in RAS (ν_imag_ = 1351*i* cm^−1^), which increased to 31.1 kcal mol^−1^ in SEL (ν_imag_ = 1390*i* cm^−1^). The latter suggests a moderately higher reactivity for RAS in the aqueous solution, which would be different from the trend observed within the MAO B active site. Still, it is very likely that a much simpler and highly polar aqueous environment favors hydride abstraction from a system with a more polar secondary amine moiety in its immediate vicinity, as in RAS, than with a more hydrophobic tertiary amine, as in SEL. Nevertheless, the thermodynamic picture of the investigated reaction is in line with the expected situation in the enzyme, as the overall reaction free energy was, by ΔΔ***G***_R_ = 2.1 kcal mol^−1^, more favorable for SEL. Subsequently, the same reacting system was immersed in the explicit aqueous solution and submitted to the free-energy calculations with the Q5 software, in order to derive the off-diagonal coupling term (H*_ij_*) and the solution-phase shift (α_0_) for each inhibitor (see the Materials and Methods section later), to be utilized in the corresponding simulations within the enzyme environment (Table 1).

**Figure 3:**
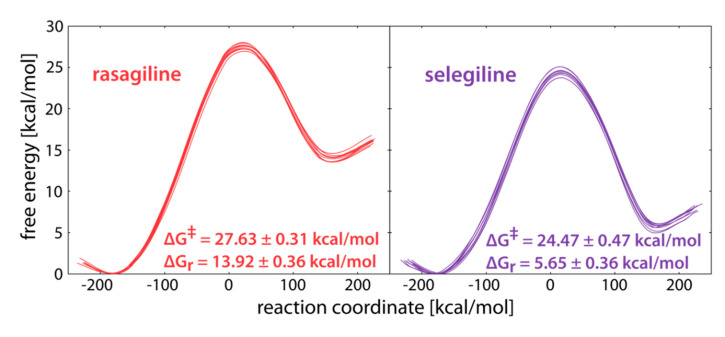
Free energy profiles for the irreversible inhibition of MAO B with rasagiline (**left**) and selegiline (**right**). The reaction coordinate is defined as the energy difference between EVB states 2 and 1 and is commonly used in displaying EVB free energy profiles.

The reaction profiles for the reaction in the MAO B active site are presented in Figure 3. Given that each profile consisted of 10 distinct trajectories, corresponding to different initial inhibitor configurations, we can safely state that all performed simulations were well-converged and pointed to consistent conclusions. The calculated activation free energies of Δ***G***^‡^(RAS) = 27.6 and Δ***G***^‡^(SEL) = 24.5 kcal mol^−1^ indicated a higher reactivity of SEL, being in excellent agreement with the determined ***k***_inact_ values of 0.99 min^−1^ for SEL [71] and 0.0533 min^−1^ for RAS [72]. Even more so, these experimental parameters translated to a difference in the activation free energy of 1.7 kcal mol^−1^ in favor of SEL, which was nicely matched by our calculations of ΔΔ***G***^‡^ = 3.1 kcal mol^−1^ here, thus lending to a strong credence to the presented results. In line with that, the calculated reaction free energies also favored the inhibition reaction with SEL, as the hydride abstraction was significantly more exergonic with that inhibitor (ΔΔ***G***_R_ = −8.2 kcal mol^−1^). Interestingly, these value were between 5 and 12 kcal mol^−1^ more exergonic than those in water (Table 1), suggesting that the charged intermediates formed upon the H^–^ transfer, were better stabilized within the enzyme than in the aqueous solution, as already seen with several MAO substrates [55,56,57,58].

**Figure 4:**
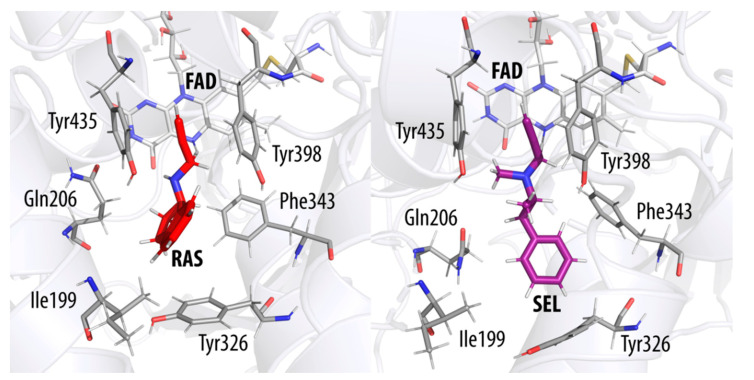
Transition state structures of the MAO B active site with the reacting rasagiline (RAS, **left**) and selegiline (SEL, **right**). The flavin cofactor is denoted by FAD. The transferring hydride ion is located about halfway between the reactive C(α) atom of each inhibitor and the N5 atom on flavin.

On the other hand, if we consider the calculated activation free energies between the aqueous solution and the enzyme environment, we observe that MAO B lowered the inactivation barrier by 2.97 kcal mol^−1^ for RAS, and as much as 6.64 kcal mol^−1^ for SEL (Figure 4). By employing the transition state theory, the corresponding increase in the reaction rate constant could, therefore, be estimated to between 2 and 5 orders of magnitude at room temperature, meaning that the reaction within the enzyme proceeded significantly faster relative to the reaction in water. To put these numbers in a proper context, let us mention that our earlier work demonstrated that the catalytic effect of the MAO B enzyme was much larger—by 9 orders of magnitude for the MAO B catalyzed dopamine degradation [56], relative to the same process in water, which is reasonable given that the structure of the enzyme is evolutionally optimized for its physiological catalytic role, rather than for its irreversible inactivation. In this context, it is interesting to note that the active site of MAO B facilitated the inhibition reaction with SEL much more, which was able to make use of the enzyme environment to overcome its lower intrinsic hydride-abstraction tendency over RAS, as evidenced with results in a pure aqueous solution (Table 1). This again confirms an earlier observation [52] that, for a successful design of novel inhibitors based on the propargylamine functionality, it is likely to be beneficial to have a tertiary instead of a secondary amino group within the structure.

Lastly, let us reiterate that a full chemical process leading from bound inhibitors to the final inhibitor–FAD complex involves three steps [52], which would all need to be considered if a complete thermodynamic picture is desired. Still, the insight obtained here for the rate-limiting first step is already very informative and illustrative, while providing important guidelines in the design of more effective and potent MAO irreversible inhibitors.

## 3. Materials and Methods

To calibrate our Empirical Valence Bond simulations of the enzyme inhibition reaction, we performed the corresponding simulation of the nonenzymatic hydride transfer reaction in the aqueous solution, which represented the reference process. The tunable EVB parameters, namely the relative shift of potentials representing the reactant and product valence states (α_0_) and the coupling parameter between these valence states (H*_ij_*), were obtained by fitting to the activation (Δ***G***^‡^) and reaction (Δ***G***_R_) free energies obtained from the QM calculations in water solution, which is a valid approach due to the demonstrated phase-independence of the EVB parameters [73]. The fitted EVB parameters were then applied to the reaction simulation carried out in the fully featured environment of the solvated MAO B enzyme (see below) to yield the free energy profiles presented here.

For the mentioned QM calculations in water, the structure of the FAD cofactor was truncated to the lumiflavin (LMF) moiety (Figure 2, with the –CH_2_–S–Enzyme fragment replaced by –CH_3_), while SEL and RAS were considered in full. The QM reaction energetics were calculated using the M06–2X/6–31 + G(d,p) level of theory with the SMD implicit solvation employing parameters for pure water, also used to obtain thermal corrections without the scaling factors, so that all computed values corresponded to Gibbs free energies at a room temperature of 298.15 K and a normal pressure of 1 atm. The validity of the obtained transition state structures was confirmed by the frequency analysis (ν_imag_ = 1351*i* cm^−1^ for RAS and 1390*i* cm^−1^ for SEL) and the intrinsic reaction coordinate (IRC) calculations. The reaction free energy was calculated as the difference between the energy of the reactants complex and the transient intermediate point on the products reaction coordinate path in which the hydrided LMFH^−^ moiety remained planar, in line with our previous reports [56,74,75,76]. All QM calculations were performed using the Gaussian 16 program package [77].

**Figure 5:**
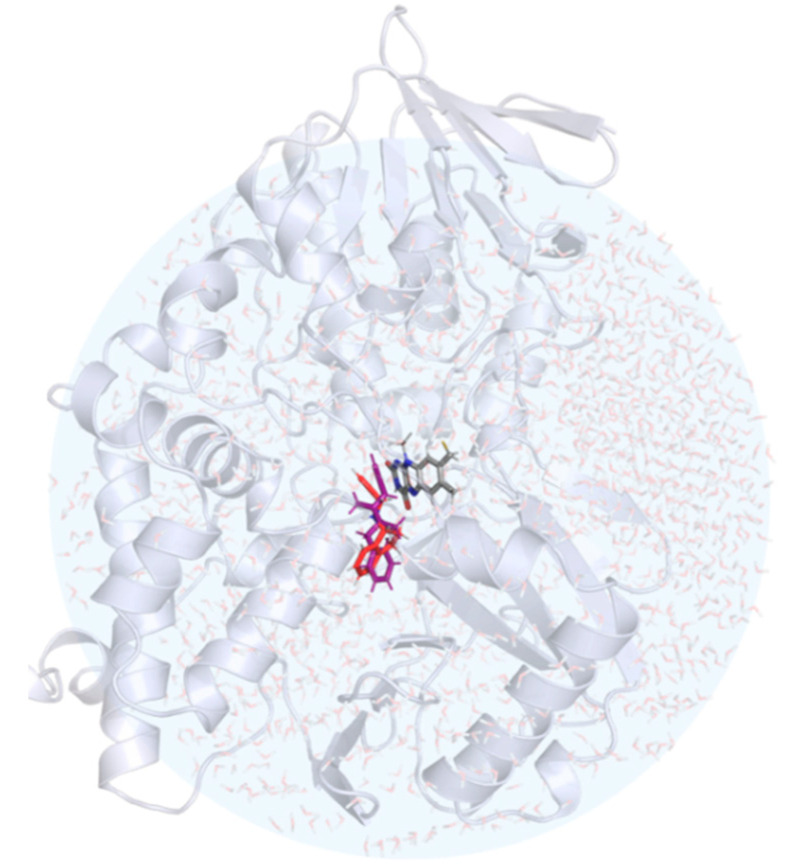
The structure of the hydrated MAO B with rasagiline (in red) and selegiline (in violet) placed in the active site. The position of the FAD cofactor is also shown in the stick representation.

The starting points for our EVB simulations were the coordinates of the MAO B enzyme in complex with the bound NYP inhibitor (PDB ID: 1GOS) [34]. The inhibitor was removed, but its position in this structure served as a reference point for the initial manual positioning of RAS and SEL into the active site (Figure 5) using the UCSF Chimera program [78]. The protein model included one subunit of the dimeric MAO B enclosed in a simulation sphere, with a 30 Å radius, centered at the reactive N5 atom of the FAD cofactor. Such a setup encompassed the vast majority of the protein—either RAS or SEL—and 1662 TIP3P water molecules. All protein atoms outside this sphere were kept restrained to their starting positions by applying a 200 kcal mol^−1^ Å^−2^ harmonic restraint. The simulations were built around the OPLS-AA force field [79], with the ligand parameters acquired by the ffld_server utility and assisted by the Maestro v. 11.7 graphical interface [80]. The charges of the ligand atoms were determined by fitting to the electrostatic potential computed by QM calculations on the HF/6–31G(d) level of theory according to the RESP scheme, as implemented in AmberTools18 [81]—all in line with our previous reports [56,57,58,69,70,74,75,76,82,83].

The system was first equilibrated in several distinct steps, by slowly increasing both the temperature (starting at 1 K and ending at 300 K) and the time-step (from 0.1 to 1 fs), as well as gradually removing the restraints. An additional equilibration step of 10 ns was carried out at 300 K with minimal position restraints. Such an equilibrated structure was used as the starting point for the subsequent simulations, which employed standard EVB procedure based on the free energy perturbation/umbrella sampling (FEP/US) approach [67,84,85]. In the case of FEP, the force fields which describe the valence states of reactants and the products (Figure 2) must first be established. This force field was appropriately tuned to allow for the breaking and formation of bonds, by replacing the harmonic potentials of the C–H and N–H bonds with Morse functions, as well as substituting the 12-6 Lennard-Jones potential with a less restrictive Buckingham-type nonbonding potential on the three reacting atoms. The reactants were then converted to the products in a series of mapping steps, using a mapping potential of the type [84]:

ε_m_ = λ∙ε_1_ + (1 − λ)∙ε_2_

where the force field of the reactants (ε_1_) was gradually transformed into the force field of the products (ε_2_) via the coupling parameter lambda (λ). In our case, the initial structure was equilibrated at λ = 0.5 (i.e., a structure in the vicinity of the transition state). Thus, the subsequent FEP procedure was carried out starting at λ = 0.5 and finishing at either λ = 0 or λ = 1, corresponding to reactants or the products of the hydride abstraction reaction (Figure 2), and yielding 51 mapping steps. Each mapping step was 100 ps long, totaling in 5.1 ns. Since the simulations were carried out in 10 independent replicas, corresponding to different initial conformations of each inhibitor (each of which was first additionally equilibrated for 100 ps), this gave us 51 ns of the total simulation time. All simulations were subject to a cut-off of 10 Å for nonbonded interactions, except for the atoms in the EVB region (RAS or SEL and the flavin moiety), which were subject to a much extended cut-off of 99 Å. Beyond that point, the electrostatic interactions were treated with the local reaction field method. All FEP simulations employed minimal restraints, necessary to obtain smooth free energy profiles, as discussed in our previous studies [82,83], including a position restraint of 0.5 kcal mol^−1^ Å^−2^ within the EVB region, with an additional restraint on the distance between the reacting inhibitor carbon (Cα) and FAD nitrogen (N5) atoms (5.0 kcal mol^−1^ Å^−2^ for distances greater than 3 Å).

A similar protocol was employed to generate EVB simulations in the aqueous solution, where the equilibration runs were somewhat shorter, due to the smaller complexity of the environment compared to the enzyme. The spherical simulation cell consisted of 3768 and 3762 water molecules for RAS and SEL, respectively, and included the truncated lumiflavin moiety (LFN). The starting structures for all simulations were generated using the *qprep5* module, while all EVB molecular dynamics simulations were carried out using the *qdyn5* module of the Q program v. 5.06 [86], with its *qfep5* module used for the analysis of the trajectories and the VMD program [87] for their visualization. All calculations were carried out at the Ažman Computing Center of the National Institute of Chemistry (Ljubljana, Slovenia).

## 4. Conclusions

In this work, we performed a multiscale computational analysis of the initial rate-limiting step of the irreversible inhibition of the MAO B enzyme by clinical inhibitors rasagiline and selegiline, involving the hydride anion transfer from the inhibitor C(α) atom to the flavin co-factor N5 atom, by using the state-of-the-art Empirical Valence Bond (EVB) QM/MM treatment. By properly including fully featured solvated enzymatic environment, well converged free energy profiles were obtained that gave the activation free energies of Δ***G***^‡^(RAS) = 27.6 and Δ***G***^‡^(SEL) = 24.5 kcal mol^−1^. The computed values underline a higher reactivity of SEL, being in excellent agreement with the ***k***_inact_ data and the experimentally determined ΔΔ***G***^‡^ = 1.7 kcal mol^−1^ that point to the same conclusion. In line with that, the calculated reaction free energies also favor the inhibition reaction with SEL, as the hydride abstraction is significantly more exergonic with that inhibitor (ΔΔ***G***_R_ = −8.2 kcal mol^−1^).

The obtained results strongly support the validity of the proposed hydride ion transfer as the likely mechanism for the irreversible inhibition of MAO B with propargylamine inhibitors. In this way, the acquired insight firmly confirms that both rasagiline and selegiline are mechanism-based inhibitors, being strongly in line with experiments. The present study represents one of only a few attempts to elucidate a molecular basis for the inhibition pathway of MAO enzymes, which bears a significant relevance for neurology. In addition, the discussion presented here offers guidelines for a rational design of new and more effective inhibitors, which are all clinically employed in treating a variety of neuropsychiatric and neurodegenerative conditions. Additionally, such improved transition state analogues are likely to be associated with less adverse effects, thus allowing for lower daily doses, tying in with some of the most prominent challenges of modern medicine.

## Figures and Tables

**Table 1:** Relative Gibbs free energies for the relevant states of the reference aqueous-phase hydride abstraction reaction among rasagiline or selegiline and lumiflavin calculated at the (SMD)/M06–2X/6–31 + G(d,p) level of theory. The derived EVB off-diagonal coupling term (H*_ij_*) and the solution-phase shift (α_0_) are also presented. All values are in kcal mol^−1^.

Inhibitor	Reactant Complex	Transition State	Intermediate	H*_ij_*	α_0_
Rasagiline	0.0	30.60	19.18	44.34	106.7
Selegiline	0.0	31.11	17.29	43.59	80.40

## Abbreviations

AD	Alzheimer’s disease
DFT	density functional theory
EVB	empirical valence bond
FAD	flavin adenin dinucleotide
FEP	free energy perturbation
LRF	local reaction field
MAO	monoamine oxidase
MM-GBSA	molecular mechanics-generalized Born and surface area
PD	Parkinson’s disease
QM	quantum mechanics
QM/MM	quantum mechanics/molecular mechanics
RAS	rasagiline
SEL	selegiline
US	umbrella sampling
